# A Non-randomized Controlled Trial of EMDR on Affective Symptoms in Patients With Glioblastoma Multiforme

**DOI:** 10.3389/fpsyg.2018.00785

**Published:** 2018-05-22

**Authors:** Monika Szpringer, Marzena Oledzka, Benedikt L. Amann

**Affiliations:** ^1^Faculty of Medicine and Health Sciences, Jan Kochanowski University, Kielce, Poland; ^2^Institut de Neuropsiquiatria i Addiccions, Hospital del Mar, Centro Fórum Research Unit, Centro de Investigación Biomédica en Red de Salud Mental, Barcelona, Spain; ^3^IMIM (Institut Hospital del Mar d’Investigacions Mèdiques), Barcelona, Spain; ^4^Department of Psychiatry, Autonomous University of Barcelona, Barcelona, Spain

**Keywords:** EMDR therapy, brain cancer, coherence, anxiety, depression, anger

## Abstract

Glioblastoma multiforme (GBM) is a highly aggressive brain cancer and its survival after diagnosis is less than 2 years. Therefore, GBM patients are especially prone to co-occurring psychological conditions such as anxiety and depressive disorders. Furthermore, aggressive medical therapies affect patients’ lives, undermining their sense of meaning and coherence. The main aim of this study was to determine the effectiveness of Eye Movement Desensitization and Reprocessing (EMDR) therapy on anxiety, depression and sense of coherence in patients with GBM. Thirty-seven GBM-diagnosed women were included in this trial and received standard medical care. Of those, 18 patients were treated during 4 months with 10–12 individual EMDR sessions (60–90 minutes each). Nineteen GBM patients were used as a non-randomized control group as they consented to psychological evaluations but not to a psychotherapeutic intervention. The groups were homogeneous in terms of gender, age, educational level and treatment, but not in anxiety and depressive levels at baseline. All patients were evaluated at baseline, after treatment (4 months) and at follow-up (further 4 months) by the Hospital Anxiety and Depression Scale (HADS-M) and the Sense of Coherence Scale (SOC-29). Caregivers in both groups were interviewed by the Patient Caregiver Questionnaire after 4 months follow-up. Statistical analyses were conducted using ANOVA statistics, correlation and regression analysis. Results showed a statistically significant decrease in the EMDR group in anxiety, depression and anger, when compared to the experimental group. EMDR therapy also had a positive impact upon the sense of coherence level in the experimental group, whereas in the control group this declined. Finally, the caregivers reported beneficial outcomes of the EMDR therapy with less anxiety- and anger-related behaviors in patients in the experimental group compared to the control group. This study is the first to show beneficial effects of EMDR therapy in alleviating affective symptoms and improving coherence in a severe medically ill population with GBM.

## Introduction

Cancers of the brain are among the greatest challenges of today’s medicine. Brain tumors, which are the most difficult to treat, are included in the Grade 4 group of cancers and are determined as high grade glioma (HGG) (Woehrer et al., 2013). Glioblastoma multiforme (GBM) belongs to this group and is the most malignant. It is responsible for around 3–4% of the mortalities among cancer patients (Carlsson et al., 2014; Razavi et al., 2016), with an average survival after diagnosis of approximately 15–17 months (Li et al., 2010; Huang et al., 2017). Only 5% of patients survive 5 years from diagnosis (Carlsson et al., 2014). Treatment strategies such as surgical intervention, chemotherapy, radiotherapy or steroid therapy with their well-known side-effects represent a further burden for the patients beyond the diagnosis.

As a consequence, anxiety and depressive symptoms appear frequently and are a widely occurring reaction to a cancer diagnosis (Kadan-Lottick et al., 2005; Kandasamy et al., 2011; Andersen et al., 2014; Sharpe et al., 2014; Lyon and Wang, 2016). Given time, these affective symptoms usually result in a major depressive disorder (MDD) or anxiety disorder on long-term (Archer et al., 2012; Pereira et al., 2012; Salvo et al., 2012). A meta-analysis of 62 studies conducted by Pinquart and Duberstein (2010) demonstrated that depressive symptoms could result in a diagnosis of MDD over time in patients with various types of cancer. Interestingly, a study conducted by Pelletier et al. (2002) demonstrated that intensification of depressive symptoms in patients with brain tumors is even significantly higher than in patients with other types of cancer. Approximately 40% of examined patients were diagnosed with MDD, whereas this was true in 15 to 30% in the case of other cancer types. Other studies of brain cancers indicate that depressive disorders affect 15–38% of patients (Pangilinan et al., 2007), with 28% patients fulfilling diagnosis of MDD (Wellisch et al., 2002). Furthermore, it has been proposed that in case of a GBM diagnosis, subjects experience this as a severe traumatic, life-threatening event which influences the meaningfulness, comprehensibility, and manageability of their lives, defined as “sense of coherence” by Antonovsky (1979). Thus, appropriate psychological assistance and psychotherapy should accompany subjects recently diagnosed with GBM. One potential therapeutic option is Eye Movement Desensitization Reprocessing (EMDR) therapy which was developed by Francine Shapiro almost three decades ago for the treatment of post-traumatic stress disorder (PTSD). The therapy aims, via bilateral stimulation, to reprocess traumatic memories through reinterpretation and inclusion in the existing memory network, using an eight-phase EMDR protocol (Shapiro, 2002; Boukezzi et al., 2017). The efficacy of EMDR for PTSD has undergone the scrutiny of various meta-analyses (Van Etten and Taylor, 1998; Davidson and Parker, 2001; Bradley et al., 2005; Seidler and Wagner, 2006; Bisson and Andrew, 2007; Benish et al., 2008; Jonas et al., 2013; Chen et al., 2014, 2015; Cusack et al., 2016). In 2013 it was also recommended by the World Health Organization as a first line treatment of PTSD (World Health Organization [WHO], 2013). So far, three pilot studies have investigated the effect of EMDR in oncological patients suffering from various types of cancer (Capezzani et al., 2013; Faretta et al., 2014; Jarero et al., 2015) but none have included GBM patients.

The present trial aimed to study, for the first time in a controlled design, the effect of EMDR therapy on anxiety, depression and sense of coherence in a sample of female patients suffering from GBM. The hypothesis of this trial was that GBM patients would improve with EMDR in affective symptoms and sense of coherence when compared to the control group.

## Materials and Methods

### Ethics Statement

The study was approved by the Bioethics Committee at the Faculty of Medicine and Health Sciences in Kielce and all patients signed an informed consent and agreed to participate in the study.

### Participants

The study included 37 GBM patients and their 37 caregivers, coming from Warsaw in Poland. All patients were outpatients and had received at baseline steroid therapy. Once included in the study all patients were additionally treated with radio- and chemotherapy. None of the patients fulfilled indication for a surgical intervention. The time between diagnosis of GBM and study entry was in all cases between 2 and 3 months. None of them had received psychological or supportive therapy before. None of the patients received psychopharmacotherapy before or during the study. Caregivers, indicated by patients as those who provided them with direct care, were also included in the study and were evaluated as a further objective source of possible psychological changes. The study participants were receiving medical care at the Oncology Centre in Warsaw and gave their consent to take part in the study. For ethical reasons, due to the high mortality of the cancer type, this study was designed as a non-randomized, controlled trial. Patient consent to receive the EMDR therapy was the condition for being assigned to a specific group The EMDR group consisted of persons who, after being diagnosed with cancer, expressed their consent to use EMDR therapy (18 patients) whereas the control group did not consent to a psychotherapeutic intervention but did to evaluations (19 patients). Both groups, however, were comparable in demographic variables such as gender, age and socio-economic status (see **Table 1**).

**Table 1:** Baseline demographic and clinical characteristics.

Characteristics	Experimental group (*N* = 18)	Control group (*N* = 19)	Statistics
**Gender**		All female	
**Age (min – max)**	63.00 (52–5 7)	65.50 (53–79)	*t* = 0.841 *P* = 0.406 (n.s.)
**Children**			
Yes	18	19	*x*^2^ = 0.094 *p* = 1.000 (n.s.)
**Education level**			
Elementary	1	0	*x*^2^ = 0.000
Secondary	10	11	*p* = 1.000 (n.s)
Higher	7	8	
**Employment at the time of diagnosis**			
Yes	13	12	*x*^2^ = 0.056 *p* = 0.728 (n.s.)
**Being in a relationship at the time of diagnosis**			
Yes	14	14	*x*^2^ = 0.000 *p* = 1.000 (n.s.)

The following in- and exclusion criteria were applied:

(1) diagnosis of a GBM brain tumor; (2) did not qualify for surgical intervention; (3) was diagnosed no earlier than 3 months prior to start of the study; (4) outpatient; (5) was not receiving individual or group psychological or psychotherapeutic therapy; (6) no psychopharmacotherapy; (7) had a level of communication allowing to perform a psychotherapy, and (8) consented to participate in the study.

### Measurements

As primary outcome criteria we explored anxiety and anger symptoms of the patients using the self-rating Hospital Anxiety and Depression Scale (HADS-M) questionnaire (Zigmond and Snaith, 1983, validated in Polish by Majkowicz, 2000). The following thresholds are defined for both depression and anxiety: 0–7 (no disorder); 8–10 (boundary state); 11–21 (confirmed disorder). The original version consists of 14 items which was expanded to 16 items in the validated version (from 0–3). Two items evaluate anger, proposing the higher the result obtained by the examined person, the higher the level of anger currently experienced by the patient. The α-Cronbach’s α coefficient for the modified questionnaire was 0.887 (Majkowicz, 2000). Of note, this scale is an evaluation of symptoms but not a diagnostic interview.

Furthermore, caregivers were assessed with respect to possible affective changes. The caregivers’ assessments were analyzed based on results obtained from the Patient Caregiver Questionnaire. This questionnaire was developed based on a pilot study of 100 randomly selected persons, who had cared for GBM patients for at least 5 years (publication in process). They provided information on the most characteristic psychopathological changes with a focus on the expression of anger or anxiety. The results obtained were ordered from the most to the least frequent in the descriptions provided, and the six most common for each group were selected. Consequently, the Patient Caregiver Questionnaire was developed by the authors of the present study, consisting of 12 questions divided into two groups: questions concerning behavior described by caregivers as anxiety-related, and questions concerning behavior described as expressing anger. Each question is assigned four possible answers, referring to the potential frequency of a given behavior’s occurrence. For each answer, the examined person is given a certain number of points from 1–4. The sum of points for each category constitutes the result, which determines the frequency of anxiety-related or anger-related behavior.

The secondary outcome criterion was the evaluation of the general psychological and emotional state of the patients, including their sense of the quality and meaningfulness of life. As mentioned before, this construct was developed by Antonovsky in 1979 and named “sense of coherence” (Antonovsky, 1979; validated in Polish, Mroziak, 1996). The self-rating Sense of Coherence Scale (SOC-29) questionnaire measures the intensity of the sense of coherence and its three components: Scom (comprehensibility); Sman (manageability) and Smf (meaningfulness). The SOC-29 questionnaire consists of 29 questions. Each question is equipped with a seven-point semantic scale, on which the examined person marks his/her answer. Evaluations of individual questions are summed up to obtain the result. The higher the result obtained on the scale, the higher is the sense of coherence. Cronbach’s α for the internal consistency of the SOC-29 questionnaire ranges from 0.84 to 0.93.

### Examination Procedure

At baseline, all participants in the study were interviewed regarding their sociodemographic data (using a questionnaire developed by the authors of the present study) and were asked to complete the before mentioned questionnaires, the HADS-M and the SOC 29. Then, patients in the experimental group started with EMDR therapy with an average length of the therapy of around 14 weeks, 12–14 therapeutic weekly sessions lasting 60–90 minutes. The standard eight-phase EMDR therapy protocol was employed by an experienced psychologist and accredited EMDR Practitioner, with a 5-year experience as an EMDR therapist. As patients were outpatients but somatically affected, EMDR therapy was performed in their homes. Fourteen weeks after baseline, patients from both groups were asked to complete the same questionnaires again. Caregivers in both groups completed also the Patient Caregiver Questionnaire both at baseline and again 14 weeks later.

### Statistical Analysis

Calculations were performed using the advanced statistical package STATISTICA 10 PL. Differences in quantitative data were demonstrated using the Student’s *t*-test for dependent samples and a Wilcoxon test. Correlation relationships between the initial and final measurements were observed using the method of series course (short series, small samples) and additionally with the Spearman’s method, due to the common ambiguity of the solutions for small samples with the use of Pearson’s method. Cohen’s *d* effect size was used for the final control of the influence of therapy on the level of anxiety symptoms in the examined patients. Correlation analyses were conducted independently for the questions asked. Qualitative observations constituted supplementary procedures. In that sense, a triangulation procedure was employed: quantitative tests were supplemented with qualitative tests of the study subject.

## Results

As regards the primary outcome, symptoms of anxiety, depression and anger decreased in a statistically significant way after EMDR therapy, when compared to the control group. Conversely, in the control group a statistically significant increase of anxiety and depressive symptoms was observed. At baseline (T0), the number of affective symptoms in the HADS-M scale in all patients examined in the experimental group indicated a confirmed disorder. After therapy, almost 25% of the patients entered in clinical remission (no disorder), while half showed a reduction of symptoms toward a boundary state, and slightly over 25% remained in the range of a disorder. In the control group, two-thirds of the sample fulfilled symptoms of a disorder and one third exhibited a boundary state. At T1 all except one patient in the control group fulfilled symptoms indicative of a disorder; only one patient had a decrease of anxiety symptoms with a sum score indicative of an absent disorder.

With respect to depressive symptoms in the HADS-M scale, at T0 almost all subjects in the experimental group exhibited symptoms indicating the possibility of a disorder. Following the application of EMDR therapy (T1), the number and intensity of depressive symptoms decreased in over 50% of the participants to the level where a disorder was absent, while almost a third remained in the boundary state and only two persons continued within the range of a possible disorder. In the control group at baseline over two thirds of the participants showed a boundary state or the absence of a disorder. At T1, the symptoms had intensified to the level of a disorder in almost all participants.

With regard to anger symptoms of the HADS-M scale, the results of the present study also indicate a significant change in the experimental group, since the intensity of the symptoms dropped by almost a half in all patients. However, in the control group a similar tendency occurred: patients in the control group demonstrated a slight decrease in the frequency of anger symptoms (**Table 2**). Baseline levels of anxiety, depression and anger differed in both groups with a statistical significant difference. Statistics can be gathered from **Tables 2**, **3**.

**Table 2:** Characteristics of anxiety, depression, and anger symptoms according to the HADS-M questionnaire in the present study, with evaluation of the variability significance.

	Experimental group (EMDR)					Control group				
	T0 (mean ± SD)	T1 (mean ± SD)	Student’s *t*	*P*	Wilcoxon test	T0 (mean ± SD)	T1 (mean ± SD)	Student’s *t*	*P*	Wilcoxon test
Anxiety	17.50 ± 2.36	9.89 ± 3.79	8.971	0.000	*p* < 0.000	13.16 ± 3.61	14.89 ± 3.25	-2.049	0.055 (n.s.)	*p* < 0.048
Depression	16.44 ± 4.03	7.56 ± 3.78	9.574	0.000	*p* < 0.000	10.79 ± 4.37	13.68 ± 3.19	-2.740	0.013	*p* < 0.016
Anger	3.39 ± 1.46	1.72 ± 0.96	4.123	0.001	*p* < 0.004	2.58 ± 1.17	2.36 ± 1.11	0.889	0.385 (n.s.)	*p* < 0.417 (n.s.)

*EMDR, eye movement desensitization and reprocessing; T0, first measurement; T1, second measurement, after therapy (in experimental group) or after 14 weeks (in control group); n.s., not significant.*

**Table 3:** Differences in levels of anxiety, depression, anger, and sense of coherence in T0 and T1.

	Experimental group (EMDR) (*N* = 18)	Control group (*N* = 19)	Student’s *t*	*P*
Anxiety – T0	17,5	13,16	4.306	0.000
Anxiety – T1	9,89	14,89	-4.324	0.000
Depression – T0	16,44	10,79	4.086	0.000
Depression – T1	7,56	13,68	-5.337	0.000
Anger – T0	3,39	2,58	1.867	0.07 (n.s.)
Anger – T1	1,72	2,36	-1.34	0.068 (n.s.)
Coherence – T0	103,278	125,579	-2.388	0.022
Coherence – T1	140,389	118,789	2.544	0.016

*EMDR, eye movement desensitization and reprocessing; T0, first measurement; T1, second measurement, after therapy (in experimental group) or after 14 weeks (in control group); n.s., not significant.*

The value of Cohen’s *d* indicated a strong relationship between the use of EMDR therapy in the experimental group and the decrease in the level of anxiety, depression, and anger symptoms (see **Table 4**).

**Table 4:** Influence of therapy on the level of anxiety, depression, and anger of examined patients.

	Experimental group (EMDR)		Control	Group
	*P*	Cohen’s *d*	*P*	Cohen’s *d*
Anxiety	0.000	2.11	0.055 (n.s.)	0.47
Depression	0.000	2.25	0.013	0.63
Anger	0.001	0.97	0.385 (n.s.)	0.20

*EMDR, eye movement desensitization and reprocessing; n.s., not significant.*

The positive result of the HADS-M scale was confirmed by the external evaluation of the caregivers of GBM patients receiving EMDR therapy. In the experimental group, a decrease in anxiety-related behavior from T0 (μ = 6.89) to T1 (μ = 3.34) (*p* = 0.021) and in anger-related behavior in T0 (μ = 5.06) to T1 (μ = 2.90) (*p* = 0.057). In change, in the control group caregivers described an increase in anxiety-related behavior from T0 (μ = 4.05) to T1 (μ = 6.31) (*p* = 0.461), as well as a slight increase in anger-related behavior T0 (μ = 4.42) to T1 (μ = 4.80) (*p* = 0.001).

The secondary outcome, sense of coherence, showed also positive results in the EMDR group. At baseline (T0) the mean sense of coherence level was lower in the experimental group (103.278; *SD* = 28.219) than in the control group (125.579; *SD* = 28.545) which resulted statistically significant (*t* = -2.388; *DF* = 35; *p* < 0.022). The same effect could be observed with regards to symptoms of depression, anxiety and anger resulting from HADS-M (**Table 3**). In T1 the mean sense of coherence in the experimental group increased (140.389; *SD* = 27.641) while it decreased in the control group (118.789; *SD* = 23.950). The difference between both was again statistically significant (*t* = 2.544; *DF* = 35; *p* < 0.016).

**FIGURE 1:**
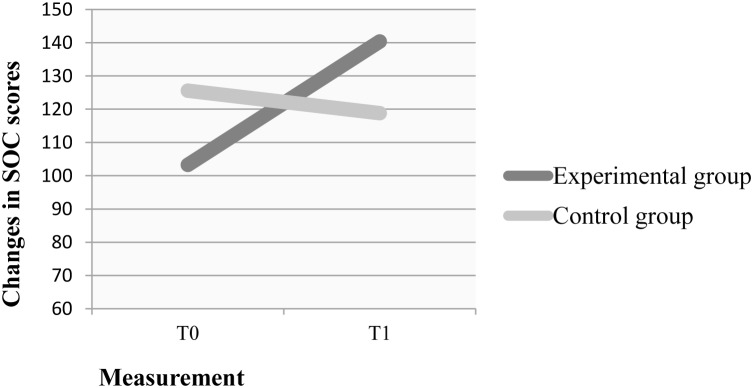
T0 - first measurement; T1 - second measurement, after therapy (in experimental group) or after 14 weeks (in control group). SOC: Sense of Coherence Scale.

The increase in the sense of coherence in the experimental group and the decrease in the sense of coherence in the control group was also statistically significant, respectively (*t* = -10.769; *DF* = 17; *p* < 0.000; *t* = 2.465; *DF* = 18; *p* < 0.024). Changes in the general sense of coherence are presented in **Figure 1**.

We also found a highly significant correlation of the general sense of coherence between T0 and T1 for the experimental group (*r* = 0.885; *p* = 0.000).

The statistics of the influence of EMDR therapy on the individual components comprehensibility (Scom), manageability (Sman), and meaningfulness (Smf) is presented in **Table 5**. The value of Cohen’s *d* demonstrates the strong influence of the EMDR therapy on all components in the experimental group. In a subsequent analysis, relationships between anxiety symptoms and the sense of coherence indicated a negative correlation, both in T0 (*r* = -0.124; *p* < 0.624) and T1 (*r* = -0.548, *p* < 0.019).

**Table 5:** Influence of EMDR therapy on the level of Scom, Sman, and Smf in examined patients.

	Experimental group (EMDR)					Control group				
	T0 (mean ± sd)	T1 (mean ± sd)	Student’s *t*	*P*	Cohen’s *d*	T0 (mean ± sd)	T1 (mean ± sd)	Student’s *t*	*P*	Cohen’s *d*
Scorn	37.94 ± 9.45	49.56 ± 10.01	-7.953	0.000	1.87	47.32 ± 7.88	40.58 ± 6.50	4.989	0.000	1.14
Sman	34.17 ± 11.67	46.00 ± 10.67	-7.008	0.000	1.65	42.47 ± 11.51	40.89 ± 9.89	1.452	0.164 (n.s.)	0.33
Smf	31.17 ± 9.45	44.83 ± 8.00	-9.555	0.000	2.25	35.79 ± 10.57	37.32 ± 9.88	-1.454	0.163 (n.s.)	0.33

*EMDR, eye movement desensitization and reprocessing; T0, first measurement; T1, second measurement, after therapy (in experimental group) or after 14 weeks (in control group; Scom, sens of comprehensibility; Sman, sens of manageability; Smf, sens of meaningfulness; n.s., not significant.*

## Discussion

To the best of the authors’ knowledge, this is the first controlled study using a structured psychotherapy, in this case the standard 8 phase EMDR protocol, in a homogenous group of patients with a specific cancer, GBM, to test whether this intervention improves psychological aspects of the disease. Overall, we found first positive evidence of EMDR on affective symptoms and sense of coherence, specifically an improvement in comprehensibility, manageability and meaningfulness, in a sample of female GBM patients. The HADS-M questionnaire was used to determine the levels of anxiety, depression, and anger and showed approximately a 50% score decrease in all patients of the experimental group after EMDR therapy.

The presence and intensification of anxiety symptoms following cancer diagnosis, as detected in all participants at baseline in our work, has been reported in previous studies. Stark and House (2000) demonstrated for instance that about 48% of 178 patients diagnosed with various cancers fulfilled the diagnosis anxiety disorders following ICD10 classification. Similar data were found in patients with breast cancer proposing a high prevalence of PTSD (Vin-Raviv et al., 2013). Of importance, EMDR reduced anxiety symptoms in our sample of GBM patients. These results are in line with three further EMDR studies conducted in subjects diagnosed from various other types of cancer which also reduced anxiety symptoms. Capezzani et al. (2013) measured for instance anxiety symptoms with the State-Trait Anxiety Inventory and the State-Trait Anxiety Inventory which decreased following EMDR therapy including patients with various types of cancers during the active phase of medical treatment. Two further studies obtained also positive results with EMDR therapy in anxiety and PTSD symptoms, respectively, in patients diagnosed also with different cancer types (Faretta et al., 2014; Jarero et al., 2015). Both Faretta et al. (2014) and Capezzani et al. (2013) studies observed also a decrease in depressive symptoms among participants following the use of EMDR therapy, measured by Back Depression Inventory (BDI) questionnaire. This positive effect of EMDR was also detected in our trial and is of importance as not only anxiety but also depressive symptoms increase over time in this population, especially if no psychotherapeutic assistance is offered (Wellisch et al., 2002; Pinquart and Duberstein, 2010).

Anger as an affective reaction to a diagnosis of cancer is understandable but understudied so far. A review by Thomas et al. (2000) concluded low levels of anger in cancer patients which were interpreted as a strong suppression and restraint of emotions considered inappropriate and reprehensible. Interestingly, at baseline (T0) we found a mean score of 3.39 in the experimental group and a mean score of 2.58 in the control group. Both can be considered as clinically relevant as scores are intermediate with the greatest intensity of 6 scores in this scale. As stated, scores in anger in our GBM sample decreased in the EMDR group but this was not statistically significant as the control group decreased as well in anger symptoms.

The positive effect on affective symptoms, especially anxiety and depression using the HADS-M, was confirmed by the Patient Caregiver Questionnaire. The differences in anger- and anxiety-related behavior in the experimental group after EMDR therapy were statistically significant. Of note, the second measurement after EMDR therapy showed that the caregivers’ assessments in relation to an improvement of anxiety and anger-related behaviors was in accordance with the subjective assessments performed by the patients themselves via the HADS-M questionnaire. The same was true for the control group where caregivers and patients both declared an intensification of anxiety-related behaviors and symptoms; however, it is interesting that anger symptoms slightly decreased as per both caregivers and patients questionnaires. These results seem relevant to us as studies of anger in cancer populations are scarce so far, especially comparing the subjective assessment of cancer patients with any kind of external assessment. Our results indicated, as stated, that in both groups the caregivers’ assessments did not differ from the assessments of the patients themselves. It cannot be excluded, though, that the caregivers’ assessment regarding anger perceived by their patients might have been in part countertransference by the caregivers via their own stress, sense of responsibility or guilt.

The present study employed also the SOC-29 questionnaire to determine the general state of patients with GBM-type cancer. This measured their level of well-being or quality of life, including their emotional state and “sense of coherence,” such as the ability to cope with situations. Numerous tools exist allowing medical practitioners to determine the well-being of cancer patients. However, as emphasized by Cheng et al. (2010), the poor physical prognosis limits typical tools for patients with cancer, including brain tumors. Analyses of the results in the present study indicate that the SOC-29 might be a useful tool as the sense of coherence increased in patients in the experimental group, both in general, and in its individual components. This finding is supported by the Cohen’s *d* value, suggesting that EMDR therapy had a strong influence on the increased levels of comprehensibility, manageability, and meaningfulness in the experimental group. At the same time, a statistically significant decrease in the sense of coherence was noted in the control group which might be due to the physical and psychological deterioration within the follow-up period.

Various other forms of psychotherapeutic assistance in the case of cancer patients (Hagerty et al., 2005; Strong et al., 2007; Espie et al., 2008; Hoffman et al., 2012) have been performed but not in a pure GBM sample. Furthermore, the majority of studies are limited solely to the determination of psychological consequences of the disease (Burgess et al., 2005; Gil et al., 2005; Linden et al., 2012). Some studies, however, focused also on the outcome of psychotherapeutic interventions and found little evidence for an improvement in affective symptoms. Breitbart et al. (2012) investigated, for example, Individual Meaning-Centered Psychotherapy in 120 patients with advanced cancer (III and IV stage), a therapy directed at methods of coping with difficult situations. They could not detect any effect of this intervention on the levels of anxiety and depressive symptoms. A similar negative result with regards to depressive symptoms was observed by de Vries et al. (1997) in an earlier study, which used individual experimental-existential counseling. A further study has been performed by Arnold et al. (2008) which evaluated the efficacy of psychopharmacological drugs in persons with brain tumors and corresponding psychopathological symptoms. Results were non-significant for psychopharmacological drugs, leading the authors to emphasize the significance and need to study psychoeducation and/or psychotherapy for this group of patients. Another study found that high drop our rates limit often psychotherapeutic interventions (Applebaum et al., 2012). In this study, more than half of the 153 patients dropped out due to a deterioration of their physical state and/or difficulties in attending the 8 programmed sessions. In light of these findings, the appropriate selection of the type of intervention gains considerable importance, particularly in patients with such a specific tumor type as GBM. In our EMDR group no patient dropped out, but our study was much smaller than the before mentioned work and candidates were well defined and in a comparable physical state at baseline.

Various limitations of our study have to be taken into account before translating our results into clinical practice. First of all, the relatively small number of included patients which limits the statistical analysis. Then, we did not randomize patients in a methodologically sound way. As stated before, this was not done due to ethical considerations, as subjects were diagnosed with a diagnosis with a high and rapid mortality. Instead, a “natural” randomization process of patients either consenting or not consenting to a psychotherapeutic intervention was chosen. The principles of random selection would indicate the use of a waiting list option. However, such an option was in our opinion not acceptable in our GBM patients, as the development of severe neurological symptoms and deterioration in communication during the study duration would have meant control group patients would afterwards have been unable to participate in a compensatory EMDR therapy. For those reasons, it was also difficult to carry out an adequate follow-up to confirm our results at mid- and long-term. Both patient groups were similar in demographic variables but a further limitation due to the lack of a randomization process is that the experimental group showed more psychopathological symptoms at baseline. This fact may suggest that the patients who granted their consent to receive the EMDR therapy were also different from the no-consent patients in terms of other psychological variables, such as sense of control, helplessness, optimism/pessimism, etc. in ways that contributed to positive outcomes in the EMDR group. Future studies could clarify this issue better by providing an alternative type of active treatment (e.g., cognitive-behavioral therapy) for the control group rather than applying no treatment at all. We also included female patients only, meaning we cannot generalize results to male patients. Finally, it is also important to emphasize that scales were self-rating evaluations which possibly created a bias in the patients’ perception of their psychological symptoms. However, the inclusion of a caregiver questionnaire added valuable and more objective information. Fidelity checks have not been performed in this study.

Strengths of the study include the pure GBM sample in a severely somatic ill population, the comparable samples in demographic variables in both groups, the use of a standardized EMDR protocol, and, as stated, patients and additional objective caregivers’ ratings. Furthermore, subjects did not receive psychopharmacological drugs as potential confounders. Finally, studies so far in this population are scarce and it is of merit and an important clinical need to include patients with a disease of a rapid and high mortality.

This study is, in our mind, an important and clinically relevant work, with the possibility that EMDR might be incorporated in oncological consultation liaison services, with the aim of improving the psychological situation of a complex population with a high somatic and psychological vulnerability. Future psychotherapeutic replication studies in GBM patients should include a larger number of patients, randomize patients possibly to a comparable psychotherapeutic intervention and scales should be hetero-applied by blind to treatment raters.

## Author Contributions

MS and MO both contributed equally to the design of the work. MO conducted the studies and collected results for the work. MS was responsible for methodical input regarding data analysis and data interpretation. MS and MO prepared the paper and approved the final version for publication. BA revised and edited the final version.

## Conflict of Interest Statement

The authors declare that the research was conducted in the absence of any commercial or financial relationships that could be construed as a potential conflict of interest.
